# Integral Role of Chest Physiotherapy and Proprioceptive Neuromuscular Facilitation in Improving the Oxygenation Index and Quality of Life of Patients With Squamous Cell Carcinoma: A Case Report

**DOI:** 10.7759/cureus.78009

**Published:** 2025-01-26

**Authors:** Samruddhi Aherrao, Lajwanti Lalwani

**Affiliations:** 1 Department of Cardiorespiratory Physiotherapy, Ravi Nair Physiotherapy College, Datta Meghe Institute of Higher Education and Research (Deemed to be University), Wardha, IND

**Keywords:** chest physiotherapy, chest proprioceptive neuromuscular facilitation, oxygenation index, squamous cell carcinoma, weaning time

## Abstract

Squamous cell carcinoma (SCC) is the most common malignant tumor of the oral cavity. Eating, speaking, and performing basic dental hygiene are among the activities significantly affected by this condition. A 48-year-old male presented with complaints of pain in his left cheek for the past three months and was subsequently diagnosed with moderately differentiated SCC of the left buccal mucosa. He underwent surgery involving composite resection of the lesion, segmental mandibulectomy, and radical neck dissection on the right side for well-differentiated SCC of the left anterior alveolar region. This case emphasizes the importance of chest physiotherapy and proprioceptive neuromuscular facilitation (PNF) in managing oral SCC. The objective is to enhance the patient's quality of life and reduce dependence on ventilator support by improving the oxygenation index. Chest physiotherapy and PNF are administered to improve chest mobility, optimize breathing patterns, strengthen respiratory muscles, and reduce secretions. In conclusion, chest physiotherapy and PNF benefit patients with oral SCC. These interventions help improve the oxygenation index, reduce weaning time, and enhance overall quality of life.

## Introduction

Oral cavity cancer is the sixth most prevalent cancer worldwide. In Europe and the United States, the tongue is the most common intraoral cancer subsite, accounting for 40% to 50% of all oral cancer cases [1]. The prevalence of oral cancer varies depending on the patient's gender and geographical location. India faces a significant risk of oral cancer due to the widespread use of cigarettes and tobacco chewing [2]. Fatigue associated with cancer is a complex issue, often arising from a combination of factors, including decreased physical activity and symptoms related to the illness or its treatment [3]. The oral cavity is uniquely located within the head and neck region. Its complex functional design enables it to perform essential roles such as speaking, swallowing, and facial expression. The anatomical oral cavity includes the mucous lip, mouth tongue, floor of the mouth, mandibular and maxillary gingiva, retromolar trigone, buccal mucosa, and hard palate subsites [4].

Lip cancer is one of the most prevalent types of cancer affecting the head and neck and is also one of the easiest to treat. Compared to other head and neck mucous membranes, the lips' exposure to sunlight significantly increases the risk of cancer [5]. The molar bone structure and neighboring soft tissues are essential for oral and facial function. The integrity of the mandible affects communication, chewing, airway maintenance, and appearance. Insufficient bone support negatively impacts all these functions [6]. Symptoms of temporomandibular disorders include headaches, jaw locking, ear, face, jaw, or neck pain, jaw popping or clicking when opening or closing the mouth, and pain while eating [7]. A limited mouth opening can affect nutrition, pronunciation, oral hygiene, treatment, and overall quality of life [8]. Physiotherapy is crucial in restoring function to the mouth, face, chest, shoulder, neck, and other areas. It is essential in preventing the side effects of chemotherapy, radiotherapy, and surgery. Physiotherapy can significantly improve the ability to speak and swallow, extend the range of motion in the neck and shoulder joints, reduce pain, enhance mouth opening, and alleviate lymphedema during the recovery phase following surgery or radiation therapy [9].

A significant portion of these patients undergo tracheostomies on an elective basis. Physical therapy is crucial in maintaining lung shape and function during the perioperative phase. Studies have demonstrated the importance of chest physical therapy for patients with tracheostomies or those on mechanical ventilation. Early mobilization and chest physiotherapy have been shown to improve extubation outcomes. Chest physiotherapy aids in airway clearance and alveolar recruitment [10]. The strength and performance of the respiratory muscles can be restored using proprioceptive neuromuscular facilitation (PNF) techniques. Consequently, it is recommended to utilize PNF approaches alongside chest physiotherapy in perioperative situations [11]. Various chest PNF techniques support sustained inspiration while improving ventilation-perfusion mismatch and the alveolar-PaO2 gradient. This reduces the likelihood of intrapulmonary shunting and acidosis [12]. This study aimed to assess the outcomes of patients with moderately differentiated squamous cell carcinoma (SCC) of the right buccal mucosa treated with chest physiotherapy and PNF, focusing on improving their quality of life and oxygenation index.

## Case presentation

Patient information

A 48-year-old man, complaining of pain over the left cheek for the last three months, presented to Siddhant Gupta Meghe (SGM) Cancer Hospital, Sawangi (Meghe), Wardha, India. The patient reported experiencing a painful, nonhealing ulcer on the left side of the hard palate, which initially appeared as a pea-sized lesion and progressed to approximately 3 x 1 cm in size. The dull, aching pain worsened during chewing and eventually subsided on its own. The pain started gradually and progressed over time. Additionally, he admitted to chewing kharra and smoking cigarettes three to four times a day for approximately 30 years. He also reported a burning sensation for the past 15 days and a weight loss of 2-3 kg over the last three months. The patient presented to the SGM ICU complaining of dull, aching, intermittent, and throbbing pain that worsened during chewing but alleviated spontaneously at times. A contrast-enhanced computed tomography (CECT) of the oral cavity was performed to aid in diagnosis. Examination revealed moderately differentiated SCC in the left buccal area. Jaw pain intensified when the patient opened his mouth or ate, but avoiding strenuous jaw movements provided relief. The patient underwent surgery and was admitted to the SGM ICU on a controlled mechanical ventilator. A physiotherapy consultation was initiated, and the patient is currently undergoing rehabilitation.

Clinical findings

Written and verbal consent from the patient's relatives was obtained. During the assessment, the patient was observed in a prone position, with the head supported on a pillow, the elbows and shoulders in a neutral position, and both knees flexed. A tracheostomy procedure had been performed, and the patient was on continuous mandatory ventilation with a positive end-expiratory pressure of 10 cmH2O and 50% FiO2. Upon observation, a Ryle's tube, drain, Foley's catheter, and IV infusion were present. The cardiorespiratory examination revealed the patient's vital signs as follows: a pulse rate of 84 beats per minute, a respiratory rate of 18 breaths per minute, and an arterial pressure of 120/90 mmHg. Following the procedure, cancer was identified in the patient's right buccal mucosa. Appropriate actions were taken. The oxygenation index, calculated using the formula (mean airway pressure × FiO2 × 100) / PaO2, was determined to be 42%. A quality-of-life assessment was conducted using the EORTC QLQ-H&N35 scale, with the patient scoring 61, indicating moderate pain experienced during the past week. Detailed documentation of the weaning trials, including start and stop times, patient progress, and any complications, was maintained.

Radiological findings

**Figure 1:**
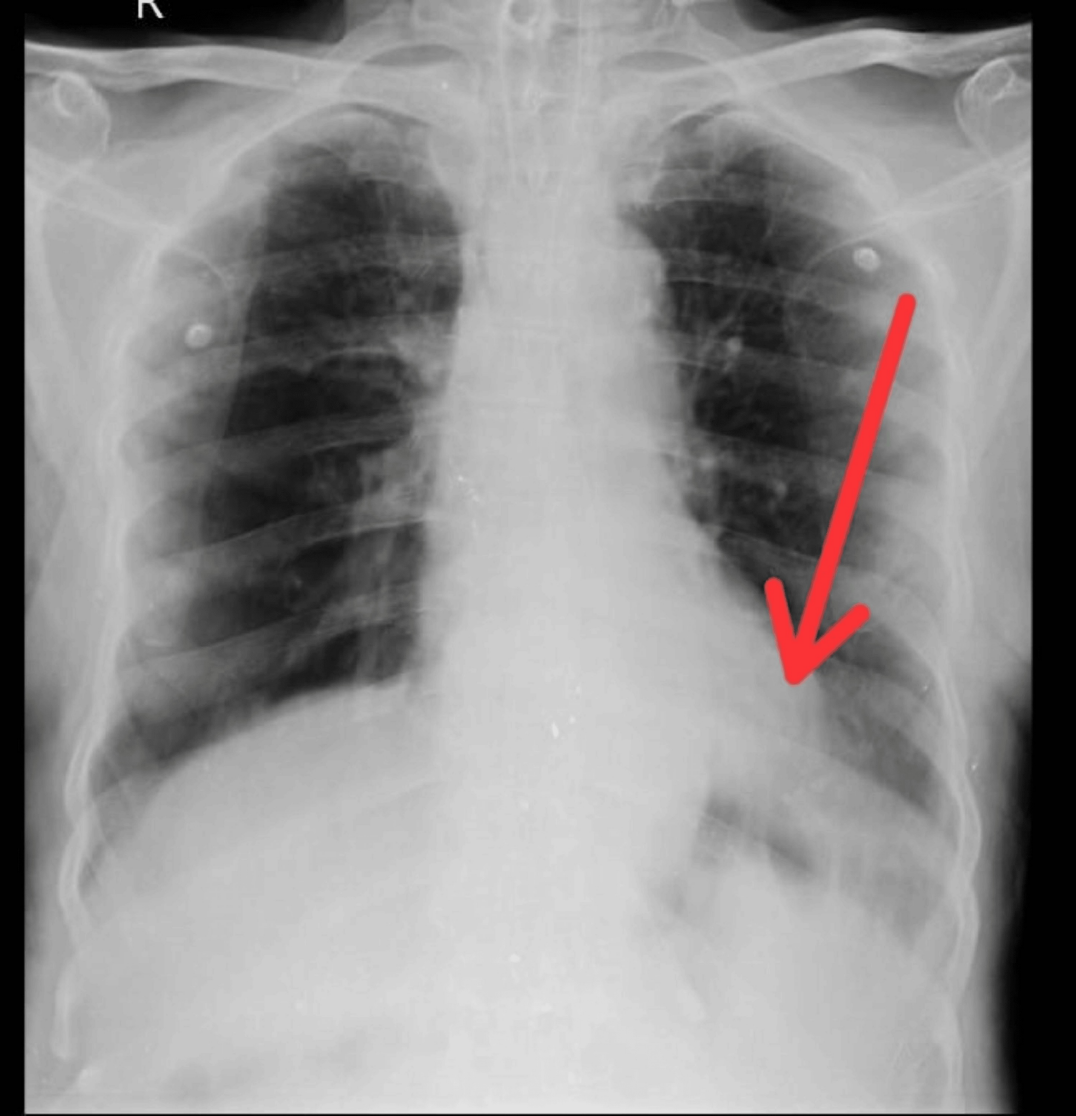
X-ray findings showing the bronchovascular markings present in the left lower zone and the right middle zone

The X-ray of a 48-year-old male reveals bronchovesicular markings present over the left lower zones and right middle zones, as shown in Figure 1.

**Figure 2:**
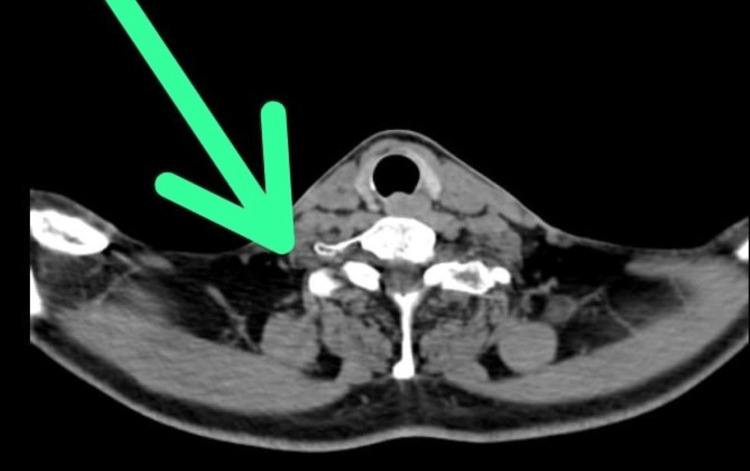
A mildly enhancing solitary soft tissue density nodule with spiculated margins, measuring 5.1 × 3.8 mm, noted in the anterior segment of the right upper lobe

In Figure 2 below, CECT reveals a mildly enhancing solitary soft tissue density nodule with spiculated margins measuring 5.1 × 3.8 mm in the anterior segment of the right upper lobe.

Timeline of events

The patient has undergone a surgical procedure for composite resection of the lesion, including maxillary alveolectomy from 21 to the hamulus, segmental mandibulectomy from 42 to the subsigmoid on the left side, and reconstruction with an anterolateral thigh flap on the left side. A tracheostomy was also performed. The timeline of events is explained in Table 1.

**Table 1:** Timeline of events according to the patient's condition

Events	Timeline
Dates of admission	29^th^ May 2024
Date of surgery	12^th^ June 2024
Date of tracheostomy procedure	12^th^ June 2024
Date of assessment	13^th^ June 2024
Date of physiotherapy rehabilitation	13^th^ June 2024
Date of wean off	19^th^ June 2024
Date of post-assessment	19^th^ June 2024

Physiotherapy intervention

In the tables below (Table 2 and Table 3), the physiotherapy intervention is explained week by week, including the problem list, goals, and intervention with dosage.

**Table 2:** Physiotherapy interventions from day 1 to week 1 N/A: not applicable, PNF: proprioceptive neuromuscular facilitation

Time	Sr. no	Problem list	Goals	Intervention	Dosage
Day 1-week 1	1	Lack of counseling	Patient caregiver education	Explain the patient's condition and the physiotherapy treatment you will start	NA
	2	Secretions present	To reduce secretions	Chest physiotherapy, i.e., percussion, vibrations, shaking, and giving over to bilateral lung zones	5 repetitions of 2 sets every 3 hours
	3	Reduced chest mobility	To improve chest mobility	PNF intercostal stretch is given, and other techniques include vertebral pressure to the upper thoracic spine, vertebral pressure to the lower thoracic spine, anterior stretch lift to the posterior basal area will be given	10 repetitions of 2-3 sets with a supine lying position with 10 seconds of stimulus pressure and 10 seconds of rest
	4	Bed sores	To prevent bedsores	Positioning is given	Every 2 hours
	5	Cough and secretions	To facilitate mucus clearance	A coughing technique is initiated to prevent further secretions	3 times a day of 10 repetitions

**Table 3:** Physiotherapy interventions from day 7 to week 2 ROM: range of motion, TMJ: temporomandibular joint dysfunction

Time	Sr. no	Problem list	Goals	Intervention	Dosage
Day 7-week 2 (as the patient gets extubated)	1	Secretions	To clear secretions and improve airway	Active cycle breathing technique	2-3 cycles every 3 hours
	2	Reduce swallowing function	To improve swallowing	Masako manoeuvre	5 repetitions of 2 sets
	3	Reduce ROM	To improve the ROM of TMJ	Jaw opening exercises, jaw closing exercises, chin tuck exercises	10 repetitions of 2 sets every 3 hours
	4	Reduce functional capacity	To restore functional capacity	Deep breathing exercises are given, which help to improve functional capacity	3 times a day of 10 repetitions
	5	Reduce functional mobility	To improve the functional mobility	Ambulation	1-2 rounds daily 2-3 times

Outcome measures

The outcome measures taken are the oxygenation index, weaning time, and the European Organization for the Research and Treatment of Cancer Quality of Life Questionnaire Head and Neck 35 (EORTC QLQ-H&N35). Pre-assessment and post-assessment are explained in Table 4 below.

**Table 4:** Pre and post-assessment of the patient's condition N/A: not applicable

Outcome	On the day of assessment treatment (13^th^ June 2024)	Post-assessment on the day of wean off (19^th^ June 2024)
Oxygenation index	42% (extracorporeal membrane oxygenation)	N/A
Weaning time	Intubate on 12^th^ June	Extubate on 19^th^ June
Quality of life	Before surgery (84), i.e., severe	After weaning (56), i.e., moderate

Oxygenation index

The oxygenation index is explained from day 1 until the patient is extubated in the table below (Table 5).

**Table 5:** Oxygenation index from day 1 when the patient is intubated to the date of extubation

Date	Oxygenation index
12^th^ June	42%
13^th^ June	46%
14^th^ June	48%
15^th^ June	52%
16^th^ June	45%
17^th^ June	35%
18^th^ June	30%

## Discussion

In this case report, the patient undergoes treatment for moderately differentiated SCC of the left buccal mucosa. Pre-assessment and post-assessment evaluations were also conducted. We conclude that chest physiotherapy helps reduce secretions and coughing, while chest PNF improves chest mobility. Thus, providing this treatment also helps enhance the quality of daily living and improves the oxygenation index. In the postoperative management of oral carcinoma, physiotherapy suggests that interdisciplinary rehabilitation should be included in the overall care of cancer survivors [12]. Numerous studies have highlighted that 80-90% of all oral malignant tumors are oral SCC [13]. Withdrawing ventilatory assistance, abruptly or gradually, is weaning off mechanical ventilation [14].

According to recent research, early mobilization benefits 70% of ICU patients, and respiratory and physical therapy interventions effectively treat several illnesses. The existing data on the efficacy of focused physiotherapy and comprehensive physiotherapy therapies were compiled in a meta-analysis to help shorten the duration of artificial ventilation and initiate the weaning process for critically ill patients. The study concludes that early chest physiotherapy interventions assist in weaning from mechanical ventilation [15]. Cancer patients often experience reduced lung function due to various factors, including tumor growth or general physical deconditioning. By incorporating chest physiotherapy and PNF into their care plans, these patients can experience improved respiratory efficiency and reduced breathing effort. Enhanced oxygen delivery to tissues is crucial for overall recovery and well-being, and there is a reduced risk of respiratory complications, such as infections. These benefits contribute to an improved oxygenation index. Thus, chest physiotherapy and PNF help improve the oxygenation index from 42% to 30%, enhancing the patient's independence. The physical and functional decline accompanying the development of PC disease is a significant obstacle to patients' quality of life. Therefore, by lowering secretions and improving respiratory patterns, chest physical therapy contributes to an enhanced quality of life [16]. Spapen et al. concluded that all chest physiotherapy techniques share the goal of reducing secretions, making it easier for them to pass through the airways. The body placement and chest movement method involves passive range-of-motion limb movement, in-bed rotations, proper chest alignment, and regular posture changes, primarily adopting an erect posture. Chest physiotherapy also aims to improve gas exchange and oxygenation through improved ventilation/perfusion matching, greater alveolar airflow, and gravitationally-based body fluid redistribution [17].

Finally, physiotherapy programs should be carefully devised, with clearly defined targets. One could also argue that the main goals of the postoperative phase are to shorten hospital stays, address pulmonary issues following surgery, and reduce the incidence of pulmonary complications after surgery. Treatment objectives for this phase include respiratory muscle training, bronchial clearance, and exercise training. Breathing exercises for bronchial clearance and lung expansion, early mobilization and ambulation, posture correction, and shoulder range-of-motion exercises should all be incorporated in the postoperative phase [18].

## Conclusions

A 48-year-old man underwent surgery on the anterolateral aspect of his thighs to repair a buccal flap on the left side. The patient recovered quickly from physical therapy, which included exercises to improve mouth opening, and he achieved this. Therefore, these exercises may be beneficial for individuals with oral SCC. Limited mouth opening makes it more difficult to maintain an open airway and significantly increases the risk of aspiration. Additionally, to clear lung secretions, blows and vibrations can be applied to each side of the chest.
